# Integrated pathway modules using time-course metabolic profiles and EST data from *Milnesium tardigradum*

**DOI:** 10.1186/1752-0509-6-72

**Published:** 2012-06-19

**Authors:** Daniela Beisser, Markus A Grohme, Joachim Kopka, Marcus Frohme, Ralph O Schill, Steffen Hengherr, Thomas Dandekar, Gunnar W Klau, Marcus Dittrich, Tobias Müller

**Affiliations:** 1 Department of Bioinformatics, Biocenter, University of Würzburg, Am Hubland, Würzburg 97074, Germany; 2 Molecular Biotechnology and Functional Genomics, Technical University of Applied Sciences Wildau, Bahnhofstraße 1, Wildau 15745, Germany; 3 Department of Molecular Physiology, Max-Planck-Institute of Molecular Plant Physiology, Potsdam-Golm, Brandenburg, Germany; 4 Department of Zoology, Biological Institute, University of Stuttgart, Pfaffenwaldring 57, Stuttgart 70569, Germany; 5 Life Sciences group, CWI, Science Park 123, Amsterdam 1098 XG, the Netherlands; 6, Netherlands Institute for Systems Biology, Amsterdam, the Netherlands

**Keywords:** Integrated network analysis, Functional modules, Metabolic profiles, Metabolic pathways, Trend test

## Abstract

**Background:**

Tardigrades are multicellular organisms, resistant to extreme environmental changes such as heat, drought, radiation and freezing. They outlast these conditions in an inactive form (tun) to escape damage to cellular structures and cell death. Tardigrades are apparently able to prevent or repair such damage and are therefore a crucial model organism for stress tolerance. Cultures of the tardigrade *Milnesium tardigradum* were dehydrated by removing the surrounding water to induce tun formation. During this process and the subsequent rehydration, metabolites were measured in a time series by GC-MS. Additionally expressed sequence tags are available, especially libraries generated from the active and inactive state. The aim of this integrated analysis is to trace changes in tardigrade metabolism and identify pathways responsible for their extreme resistance against physical stress.

**Results:**

In this study we propose a novel integrative approach for the analysis of metabolic networks to identify modules of joint shifts on the transcriptomic and metabolic levels. We derive a tardigrade-specific metabolic network represented as an undirected graph with 3,658 nodes (metabolites) and 4,378 edges (reactions). Time course metabolite profiles are used to score the network nodes showing a significant change over time. The edges are scored according to information on enzymes from the EST data. Using this combined information, we identify a key subnetwork (functional module) of concerted changes in metabolic pathways, specific for de- and rehydration. The module is enriched in reactions showing significant changes in metabolite levels and enzyme abundance during the transition. It resembles the cessation of a measurable metabolism (e.g. glycolysis and amino acid anabolism) during the tun formation, the production of storage metabolites and bioprotectants, such as DNA stabilizers, and the generation of amino acids and cellular components from monosaccharides as carbon and energy source during rehydration.

**Conclusions:**

The functional module identifies relationships among changed metabolites (e.g. spermidine) and reactions and provides first insights into important altered metabolic pathways. With sparse and diverse data available, the presented integrated metabolite network approach is suitable to integrate all existing data and analyse it in a combined manner.

## Background

**Figure 1:**
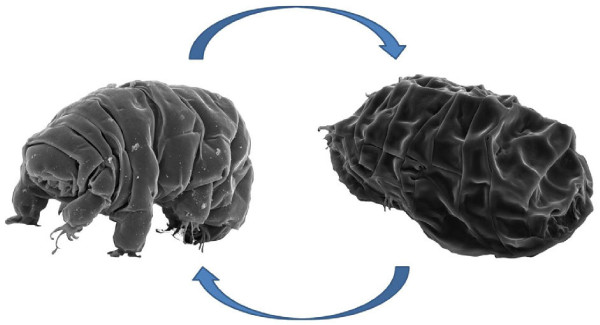
**Tardigrade transition.** The tardigrade of the species *Milnesium tardigradum* is around 1 mm in size and can be found in a diversity of habitats. In the anhydrobiotic tun state (right), tardigrades show remarkable tolerance against physical extremes. During the transition from an active into an inactive form (here by dehydration) and back (rehydration), metabolites are measured in a time series by GC-MS. The aim of the integrated analysis is to trace changes in tardigrade metabolism and identify pathways responsible for their extreme resistance against physical stress.

Tardigrades are multicellular organisms, resistant to extreme environmental changes including desiccation, freezing and radiation. They outlast these conditions in an inactive form, called tun state or cryptobiosis [1-4]. All metabolic activity decreases during tun formation up to a complete cessation of measurable metabolism until environmental conditions improve and the tardigrade returns to its active state (see Figure 1). Other invertebrate taxa that undergo cryptobiosis to escape damage to cellular structures and cell death are nematodes and rotifers [5]. All of these organisms are apparently able to prevent or repair damage under cryptobiosis. The tardigrade is a striking case as the whole animal phylum (most species) can undergo at least four types of cryptobiosis: anhydrobiosis (lack of water), anoxybiosis (lack of oxygen), cryobiosis (freezing) and osmobiosis (high solute concentration). In this analysis we investigate the metabolic mechanisms of anhydrobiosis. The tardigrade species *Milnesium tardigradum* was analysed during tun formation, which was induced by dehydration. In tardigrades few metabolites have been analysed including carbohydrates that stabilize the membrane in the dry state [6-10] or give protection and stress resistance [11-17]. The emphasis of this paper lies on the integrated analysis of metabolism during dehydration and the subsequent rehydration. To examine this, metabolites were measured in a time series by gas chromatography coupled with mass spectrometry (GC-MS). Additionally we integrated expressed sequence tags (ESTs) from *M. tardigradum* including state specific data from active and tun state [18,19]. Here we propose an integrative network approach to trace changes in tardigrade metabolism and identify pathways responsible for their extreme resistance against physical stress.

Multiple high-throughput data sets ranging from genomic over transcriptomic to proteomic data nowadays allow to analyse organisms and diseases in an integrated fashion. Interaction networks are increasingly used in current research to integrate various data sources. For gene expression profiles integrated methods are used to search for gene signatures, functional modules, subnetworks of interest or activated pathways mostly using protein-protein interaction (PPI) networks to connect the interacting partners [20-24] or by inference of regulatory networks from the molecular data [25]. Similarly to other integrated analyses, metabolic data analysis can likewise benefit from the utilisation of network information [26,27]. Technically extending the latter metabolic analysis, we characterize tardigrade metabolism using metabolite changes and EST data in the context of metabolic pathways. Similar approaches exist which integrate gene expression data, protein expression data or C13 flux data with metabolic pathways [28,29], e.g by constraining metabolic fluxes [26,30]. Usaite *et al.* integrate gene expression changes to identify connected subnetworks of enzymes with a maximum-transcriptional response in a metabolic network, this approach also accounts for highly connected metabolites in the network [31]. Others use methods based on Bayesian integration of the joint metabolomic and transcriptomic data for evaluating transcript-metabolite correlations [32] or to integrate quantitative metabolome data with genome-scale models by using a bipartite graph theoretical representation of the metabolism [33,34]. The latter two use significant changes in metabolite levels to identify reporter reactions around which the most significant coordinated metabolite changes occur.

Our analysis is based on a novel statistical approach to identify significantly changing metabolites with a trend in mass spectrometry profiles. This information is used to score the nodes of a metabolic network, constructed for this organism. Moreover, we additionally use transcriptome information to score the edges of the network. Here we implement new methods extending an exact network analysis framework heinz[21,35], which has been established for the integrated analysis of gene expression data in the context of a PPI network to calculate maximum-scoring subnetworks.

In this study we analyse the changes in metabolism, e.g. energy turnover, biosynthesis of cellular components, necessary for the change from an active state to an inactive state during dehydration and vice versa back into an active state during rehydration. Therefore, we construct a metabolic reaction network derived from the KEGG reference pathways [36], as a source for pathway information. Metabolic profiles are analysed using an Umbrella trend test, which is introduced and applied subsequently. Scores are deduced from the metabolic profiles for the nodes (metabolites) of the metabolic network. Scores for the edges of the network are derived from expression changes between the active and inactive state. These scores serve as a basis for the integrated analysis, which consists of two steps. First the metabolic network is reduced to an enriched subnetwork with enzymes and metabolites present in tardigrades. Second a maximum-scoring subnetwork is calculated from the previously enriched subnetwork using scores for differential expression and trends in metabolic profiles. The final subnetwork constitutes a functional module containing significantly altered metabolites and enzymes with expression changes between the active and inactive stage. The specific subnetwork is analysed and visualized with respect to metabolic changes which the tardigrades undergo during the transition from an active to an inactive state (dehydration) and vice versa (rehydration).

## Results and discussion

### Integrated metabolic network analysis

The combined information from several data sources was used in the context of a metabolite network to analyse changes in metabolism of the tardigrades. The strength of our approach is the integration of distinct data by a network approach to identify significant regions of interest in the network. The data used for integration consists of metabolite profiles from mass spectrometry, EST data from previous studies with mappings to EC numbers, as well as EST libraries for the active and inactive state of the tardigrades (referred to as dESTs).

**Figure 2:**
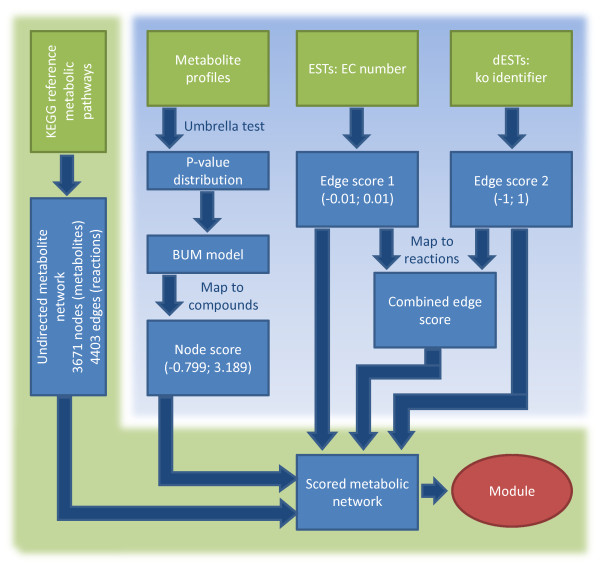
**Data integration.** Different data sources are integrated to identify a functional module explaining the metabolic changes in *Milnesium tardigradum* during de- and rehydration. On the left hand side the metabolite network is created from KEGG reference pathways. On the top of the right side different sources of molecular data are integrated: the metabolite profiles and the two sets of ESTs. From these node and edge scores are derived which are subsequently used to score the metabolic network. In the last step a maximum-scoring subnetwork is calculated using the heinz algorithm.

The overall integration process is depicted in Figure 2. It is divided into three major parts, (i) the network generation, (ii) the processing of the metabolite and EST data for node and edge scoring and (iii) the calculation of a maximum-scoring subnetworks (functional module).

#### Construction of a metabolic reaction network

A metabolic reaction network was created on the basis of KEGG reference pathways [36] and converted into a graph with metabolites as nodes and reactions as edges (see Methods). The graphs resulting from each metabolic pathway were combined into one supergraph. Subsequently, this graph was transformed into an undirected graph and pool metabolites were removed which form non-specific shortcuts in the network (see Methods for removed metabolites). The resulting network consists of 3,658 nodes which represent the metabolites and 4,378 edges representing the reactions. The nodes and edges of this network were scored in the following with the integrated data to identify a subnetwork specific for the metabolic changes in the tardigrade *M. tardigradum*.

#### Analysis of metabolite profiles

**Figure 3:**
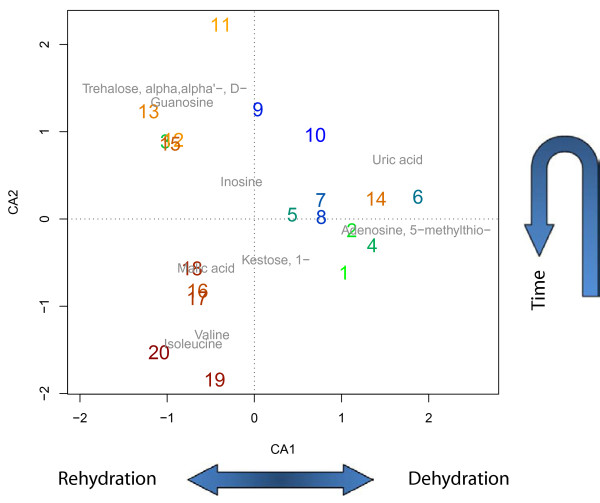
**Correspondence analysis.** Correspondence analysis (CA) of the 10% metabolites with the largest variance out of 84 metabolic profiles measured in total. Displayed are the twenty measurement time points during dehydration (from green to dark blue) and rehydration (orange to red). The first component clearly separates the two processes (de- and rehydration), while the second component separates early and late time points.

The metabolic profiles contain time course data for 84 metabolites after removal of unidentified metabolites, which were measured by GC-MS, but could not be assigned explicitly to a specific metabolite in the network. Two distinct phases of tardigrade adaptation were measured, the dehydration phase (10 time points) and rehydration phase (10 time points). Fellenberg [37] introduced correspondence analysis (CA) to identify principal factors in microarray data. In a similar manner, we applied the CA to metabolite profiles with the highest variance (top 10%, top 50% and all) to obtain the principal factors which contain most information (Figure 3). The first axis clearly separates the de- and rehydration process (Figure 3, green to blue: dehydration; orange to red: rehydration), while both first axes capture well the pattern of the metabolic time course. The CA not only allows to visualize the time points, but also the metabolites that are most specific for the axis (Figure 3, gray metabolites top 10%). The first two axis explain 66% of the total variance suggesting that the strongest signal in the metabolite data reflects their sequential change over time.

We first examined the metabolite data by testing for differences in the means of the measurements for dehydration and rehydration using a Wilcoxon test. Despite significant differences for few metabolites, a closer look at the metabolite data showed increasing and decreasing trends in the time courses. This can be explained by the slow transition from the active into the inactive stage with a cessation of metabolism at time point 10 (20 h), where 100% of the tardigrades are in the tun stage. Therefore, the experimental design required a trend test to analyse the metabolite time course data for metabolites that change most. Two methods were used subsequently, the Jonckheere-Terpstra test (JT test [38,39]) and the Umbrella test [40]. Both tests consider relative values between time points and an increase or decrease in their ranks.

**Figure 4:**
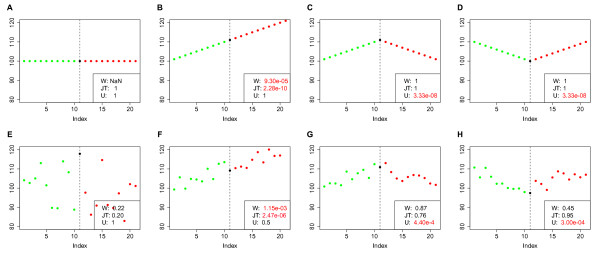
**Simulations for Umbrella and JT test.** Time series were generated containing no trend, an upward trend or a peak/low point at time point 11. **A-D** show these time series without noise and **E-H** for jittered data. On these time series the Wilcoxon, JT and Umbrella tests were performed and p-values calculated which are shown in the figure legends. Only the Umbrella test is able to identify peaked trends in the data as they are expected from the experimental design of the metabolite data.

The differences between the tests (Wilcoxon, JT test and Umbrella test) are shown for simulated time series in Figure 4 with the corresponding p-values. The JT test identifies an either increasing or decreasing monotonic trend in the data. Therefore, the resulting p-values for the JT test are only significant for a monotonic upward trend (Figure 4 B and F). All other cases do not yield significant results. In contrast to this, the Umbrella test is used to test for trends with an upward or downward apex (Figure 4 C, D, G and H). From a biological point of view it is more reasonable to consider trends with a peak, rather then monotonic trends. Since it is expected, that the metabolism changes during the dehydration (time points 1-10) and rehydration (time points 11-20) phase and should be minimal in the inactive state (time points 10-11). An umbrella form would be expected e.g. for storage metabolites or metabolites necessary for the protection of cellular structures, while for metabolites involved in energy production and cell growth an inverse umbrella shape is likely. We therefore applied the Umbrella test to the metabolite profiles and calculated the significance of peaked trends for all metabolites. The turning point of the trend was set to time point 10, where all tardigrades have completed the dehydration process.

#### Node scoring from metabolite data

**Figure 5:**
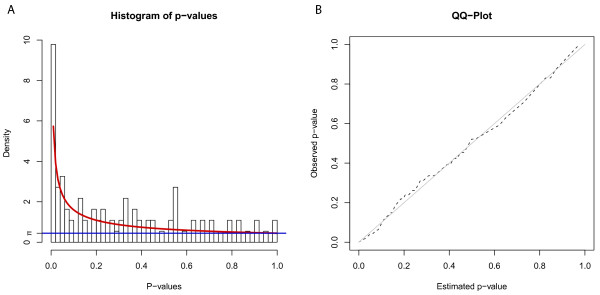
**Beta-uniform mixture model.** The fitted beta-uniform mixture model to the p-value distribution from the Umbrella test on the metabolite data is shown on the left side (**A**). The goodness of fit is shown in the quantile-quantile plot (**B**) of fitted p-values against observed p-values. *Π*denotes the *Π*-upper value which estimates the amount of noise in the data.

The p-values from the Umbrella test were subsequently used to score the nodes of the metabolite network. To convert the p-values to scores for the network and calculate functional modules the approach by Dittrich *et al*. [21] was used (see Methods). A beta-uniform mixture model (BUM) was fitted to the p-value distribution, where the beta distribution models the signal, while the noise is by definition uniformly distributed. Finally the node scores were calculated based on a log ratio of signal to noise. The fitted beta-uniform mixture model is depicted in Figure 5 with the corresponding quantile-quantile plot, validating graphically the fit of the BUM model, despite some clustering effects in the data. The *Π*-upper value of 0.4448 measures the amount of noise in the data, vice versa denoting that 55.52% of the metabolites change significantly in their profiles. The computed node scores range from -0.871 to 4.122, whereby significant p-values lead to a positive score, while non-significant p-values lead to a negative node score. Unmeasured metabolites were assigned the average of the negative scores (-0.473) which corresponds to a score derived from the noise in the data. Similarly, other methods handle unmeasured metabolites by calculating random p-values and computing scores from these [27,34].

#### Edge scoring from EST data

**Figure 6:**
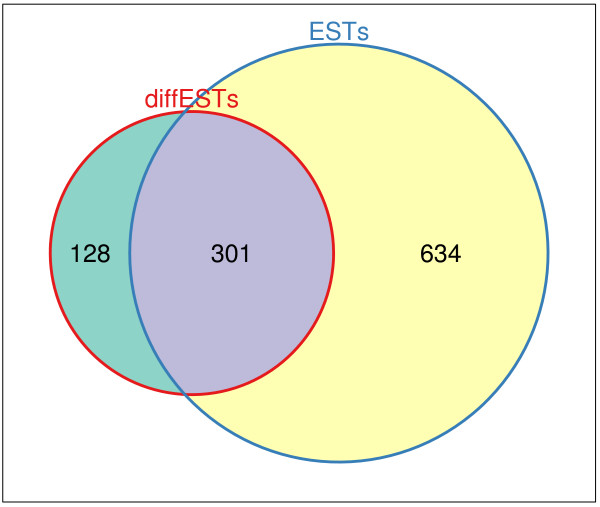
**ESTs mapped to reactions.** The Venn diagram depicts the distribution of mapped reactions to ESTs. The two sources of normal and differential ESTs cover by mapping of ko (KEGG Orthology) identifier and EC number a total of 1,063 reactions, 301 in common and 128 solely by dESTs and 634 by normal ESTs respectively.

The ESTs were subdivided into two data sets, EST data with EC number mappings from previous studies and differential ESTs (dESTs) for the active and inactive stage (see Methods). The EC number allowed the identification of corresponding tardigrade-specific enzymes and therefore reactions. The dESTs were mapped, as described in the following, to KEGG ko identifiers using KAAS [41] and thereby to reactions. The distributions of the mapped reactions to ESTs are depicted in Figure 6. The two sources of normal and differential ESTs covered by mapping of ko identifier and EC number a total of 1,063 reactions, 301 in common and 128 solely by dESTs and 634 by ESTs. The presence of ESTs mapped to an EC number were used to give a minimal weight of 0.01 to the corresponding edges (reactions). A weight of -0.01 was given to edges without identified enzyme mapping. This favoured the use of edges for which it is known that an enzyme exists, over reactions that might not exist in tardigrades, during the module search.

The dESTs were clustered into 4,422 clusters using CD-HIT-EST (see Methods). Out of each cluster a representative was used to map KEGG ko identifiers with the KEGG Automatic Annotation Server (KAAS) [41]. 898 clusters could be annotated. The log_2_ ratio of active *n*_*a*_ to inactive *n*_*i*_counts of dESTs was used for edge scoring:

(1) $$S_{e}(t)=|\log_{2}(\frac{n_{a}}{n_{i}})|-t,$$

with threshold *t*≥0 to adjust the sensitivity/specificity of *S*_*e*_similarly to the FDR used for the node scoring (see Methods, Equation 2).

#### Calculation of the metabolic module

A functional module was calculated with an exact algorithm termed heinz[21,35] using the node and edge scores to find a maximum-scoring subnetwork (see Methods). The module was calculated in a twostep approach.

First an enrichment step reduced the metabolic network to a tardigrade-specific subnetwork of 868 nodes and 1127 edges. Since the complete network was created from the KEGG reference pathways, it contains all possible metabolic reactions and metabolites of which some might not occur in tardigrades. Therefore a subnetwork was extracted based solely on the presence of enzymes and metabolites measured in tardigrades. Identified enzymes from EST data were used to score the edges and a positive node score (+1) was used for measured metabolites. Using these scores the heinz algorithm identified the maximum-scoring subnetwork, which aggregates metabolites and reactions that are likely to be present, over the positive scores.

The second step used the enriched subnetwork to score the *changes* in metabolites and *differentially abundant* ESTs. A functional metabolic module was calculated based on the node scoring from the metabolic profiles with an FDR of 0.2 (see Equation 2) and the log ratio score for the edges from the dESTs with *t*=1 (see Equation 1). The resulting edge score lay between -1 and 1, giving these edges a 100-fold higher weight than with just enzyme information (-0.01, 0.01). By integrating the different sources of information, a module was obtained representing the significant trend changes in metabolites between the dehydration and rehydration process as well as changes in EST abundance, connected by reactions for which enzymes were identified in the tardigrades.

### Physical stress-induced metabolic module for *M. tardigradum*

**Figure 7:**
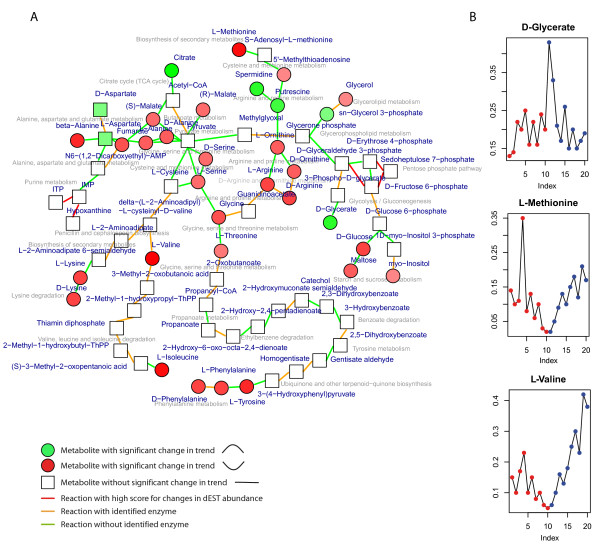
**Functional model from node scores and edge scores.** Shown is the functional module based on node and edge scores from the dESTs, calculated on a subnetwork based on edge scores from the ESTs (**A**). Circles depict nodes with positive scores, squares with negative score. The coloring of the nodes shows the Z statistic from the Umbrella test (positive: red, negative: green). Positive scores indicate an umbrella shape and negative scores an inverse umbrella shape of the time courses. For the edges the colors indicate the differential abundance of the enzyme (red), presence (orange) or absence (green) of the enzyme responsible for the metabolic reaction. Representative metabolites with a significant trend are shown in the right panel (**B**). The time points are depicted equally spaced for better visualization.

During dehydration the metabolism of tardigrades slowly reduces, up to a complete cessation of measurable metabolism in the tun stage. The recovery time during rehydration is probably a function of metabolic activities linked to repair of damage caused by dehydration and to restoration of metabolic pathways. The resulting module (Figure 7) reveals these processes by accumulating metabolic pathways involved in glycolysis/gluconeogenesis and carbohydrate metabolism, pentose phosphate pathway, the metabolism/catabolism of certain amino acids starting from pyruvate, including e.g methionine, lysine, phenylalanine, valine, arginine, tyrosine, threonine. Furthermore, trend changes in the accumulation of glycerol, which is known to protect against harmful effects of dehydration by stabilizing the membrane, is observed as well as pathways for cellular responses to osmotic stress. Significant changes in these pathways mainly show an umbrella-shaped trend in the metabolic profiles, resembling a catabolic reaction or degradation of the metabolites followed by a restoration and production of amino acids and cellular components from one-carbon sugars as carbon and energy source. These processes are also consistently identified by the GO enrichment analysis, performed on the genes represented as enzymes in the functional module (Table 1).

**Table 1:** Gene ontology enrichment of the functional module

	**GOBPID**	**Pvalue**	**Term**
1	GO:0006072	0.00006	glycerol-3-phosphate metabolic process
2	GO:0019400	0.00017	alditol metabolic process
3	GO:0009072	0.00199	aromatic amino acid family metabolic process
4	GO:0006972	0.00371	hyperosmotic response
5	GO:0006973	0.00371	intracellular accumulation of glycerol
6	GO:0071470	0.00371	cellular response to osmotic stress
7	GO:0005975	0.00652	carbohydrate metabolic process
8	GO:0006066	0.00672	alcohol metabolic process

The inverse metabolic trend with a peaked shape is less common and would be expected for storage metabolites or bioprotectants. The module includes 6 metabolites with significant changes in this direction: sn-glycerol 3-phosphate, putrescine, spermidine, aspartate, citrate and D-glycerate. Sn-glycerol 3-phosphate could potentially increase during dehydration to produce triglyceride as a highly efficient energy storage or even more likely glycerol as membrane stabilizer. Putrescine is a diamine created by the decarboxylation of ornithine. The addition of two propylamine residues yields spermidine, an essential growth factor. Other probable biological functions of spermidine are the stabilization of DNA by association of the amino-groups with the phosphate residues of the DNA, increase of RNA synthesis and enhancement of stability of tRNAs and ribosomes [42].

Especially the information on differentially abundant enzymes is valuable, by which *changes* in reactions between metabolites are identified, e.g. the link between the pentose phosphate pathway to glycolysis by the transaldolase reaction: sedoheptulose 7-phosphate + glyceraldehyde 3-phosphate *⇌*erythrose 4-phosphate + fructose 6-phosphate. Using solely the metabolite data (node scores) during the module identification these reactions would not be taken into account (data not shown). However, they seem to be highly important for tardigrade metabolism since they show strong differences in EST counts between the active and inactive state. At present only few data on the abundance of the enzymes from dESTs is available. The integrated analysis will certainly gain from the creation of additional stage-specific data to identify further important reactions and pathways.

## Conclusions

Here we present an integrated approach to analyse sparse, heterogeneous data from a largely unknown organism. For tardigrades (here: *M. tardigradum*) neither the genome is known nor the complete transcriptome or proteome, and up to now only quite incomplete analyses exist on a molecular level. This study represents a first attempt to use the existing data in an integrative approach to complement each other. By integrating metabolic profiles and transcript data into a metabolic network created from KEGG pathways, a module of concerted changes in metabolism during the process of de- and rehydration is identified.

From a statistical point of view we present a novel approach to analyse metabolite profiles. We implement the Umbrella test to identify significant non-monotonic trends in the metabolites. Furthermore, semi-quantitative differences in enzyme abundance are calculated between the active and inactive form of the tardigrade. The test results and information on enzymes are subsequently used to score the metabolic network. An advantage of our approach is the simultaneous integration of metabolite and enzyme related data into a combined analysis using node scores derived from the metabolite measurements and edge scores for differential abundance of enzymes. In contrast to previous approaches (e.g. [27]) our frame not only relies on the information of metabolic profiles, but allows the integration of molecular data for the edges of the metabolic network to model regulatory process in more detail and completeness. The resulting module thus represents metabolic pathways for which enzymes have been identified in the tardigrades, with significant trend changes in metabolite concentrations over time as well as alterations in EST abundance during the transitions between the active and inactive stage. The identified stress responsive network module mainly comprises pathways involved in glycolysis and sugar metabolism, pentose phosphate pathway and the metabolism of certain amino acids and additional reactions to store energy in triglyceride and protect the DNA.

It has to be noted, that changes in transcript abundance and metabolite levels correlate only to a certain extent. A clear relationship is evident where the metabolites are end-products (sugars, amino acids). The relationship is less transparent in those cases where the metabolites are intermediates. This has different reasons, importantly, the use of metabolic data on changes (if any) of intermediary metabolites to infer changes in metabolic rate is an intractable (possibly insoluble) problem beyond the scope of our methodology. In fact, our approach applies only statistical reasoning on more or less strongly correlated changes for different network nodes. Hence, the interpretation of the changes for the intermediary metabolites has to be done with special care. The significance levels given for our estimates are only a guiding indicator here.

Furthermore, it by now only uses a small set of measured metabolites (84) to score the nodes in the network, which is due to the limitation of current technologies. Nonetheless, is the algorithm capable of handling ’omics scale metabolic data sets, which will unquestionably be available in future studies.

A clear benefit of the presented integrative method is the identification of key processes and pathways changing during the transition phases, despite the sparsity of available data for tardigrades so far. As more data become available, including the entire genome sequence of *M. tardigradum* and more comprehensive metabolic profiles, this kind of integrated network analysis will certainly provide a powerful tool for deeper and more detailed insights into tardigrade metabolic processes and adaptations.

## Methods

### Tardigrade cultures

The study was carried out with the eutardigrade *Milnesium tardigradum* Doyère 1840, (Eutardigrada, Apochela, Milnesidae) which was originally collected in Tübingen, Germany and is since a decade as a well established laboratory culture available. The animals were cultured on petri-dishes (ø9,4 cm) with a layer of agarose (3%) (peqGOLD Universal Agarose, peqLAB, Erlangen Germany) covered with a thin layer of Volvic-water (Danone Waters, Wiesbaden, Germany) at 20°C. The animals were fed bdelloid rotifers, *Philodina citrina* (Ehrenberg, 1832), which had been raised on the green algae of the species *Chlorogonium elongatum* (Dangeard, 1897). For this study *M. tardigradum* starved over two days before harvest to avoid contamination with food-organisms. After repeated washing with clean water, animals were transferred into microliter tubes (400 individuals per tube). By using a micropipette, the animals surrounding water was reduced by careful aspiration to approximately 1.5 +/- 0.5 *μL*. The open microliter tubes were then exposed for dehydration to 85% relative humidity (RH) in a small chamber containing a saturated solution of KCl (Roth, Karlsuhe, Germany). The first time point of the dehydration period was sampled and frozen in liquid nitrogen after 1 h. Further samples were taken after 720 min, 900 min, 1020 min, 1080 min, 1110 min, 1140 min, 1170 min, 1185 min and finally after 1200 min after which the cryptobiotic tun formation was completed in all individuals. To produce different states of rehydration we added 10 *μL*Volvic-water into each microliter tube. The first rehydration state was immediately sampled and frozen in liquid nitrogen. Further samples during rehydration were taken 5 min, 10 min, 15 min, 20 min, 60 min, 90 min, 150 min, 210 min and 270 min after adding the water.

### Metabolic analysis

Metabolic analysis was performed using GC-MS based metabolite profiling [43] of a whole organism extract with modifications for automated GC-time of flight (TOF)-MS application [44]. Pools of 400 *M. tardigradum* individuals were homogenized by sonification on ice with 150 *μ*L 100% methanol which was pre-cooled to −20°C. Homogenized samples were extracted with an internal standard [44] for 15 min at 70°C, cooled to room temperature, re-extracted with 100 *μ*L CHCl_3_ for 5 min at 37°C, mixed with 200 *μ*L water and centrifuged 5 min at 14000rpm to separate liquid phases and to remove debris. The upper methanol/water phase was collected and dried in a speed vac. Metabolite extracts were stored and shipped as was described previously [44]. The dried metabolite extract was chemically derivatized and subjected to GC-TOF-MS analysis by sequential methoxyamination and trimethylsilylation and GC-TOF-MS profiling [44]. The GC-TOF-MS chromatograms were processed by TagFinder-Software [45]. Four technical repeats were analysed. Compounds were identified under manual supervision by matching to the reference library of mass spectra and retention indices of the Golm Metabolome Database, http://gmd.mpimp-golm.mpg.de[46]. Retention index thresholds for compound matching were according to Strehmel and co-authors [47]. Averaged normalized mass detector responses of each metabolite observed in the four technical repeats were calculated using specific and selective mass features [45]. Relative changes of metabolite pool sizes were assessed. Normalization was performed by the mass detector response of the internal standard and the number of individuals per sample. Metabolites that could not be separated on the basis of chromatographic elution patterns were eliminated from subsequent analysis. By this criteria, the initial list of 132 metabolites measured on the platform was thus narrowed to abundances for 92 metabolites. The normalized metabolic data is provided as an Additional file (Additional file 1).

### EST sanger sequences and KAAS annotation

Two EST libraries were used, first with EC numbers annotated ESTs from a current study (EST sequences available in the tardigrade workbench at http://waterbear.bioapps.biozentrum.uni-wuerzburg.de[19,48]) and second ESTs from the active and inactive state of *M. tardigradum* (dEST) available at NCBI (for details see [18], raw traces were submitted to the trace archive TI:2317005893-2317015871). Base calling on Sanger sequencing traces was performed using phred (version 0.071220.b) [49,50]. Adapters and vector sequences were masked using cross_match (version 1.090518) with parameters -minmatch 8 -minscore 20 [51] and trimmed using SnoWhite (version 1.1.3) [52]. The dEST sequences were clustered into 4,422 clusters using CD-HIT-EST (v4.3) with a similarity threshold of 90% including forward and reverse matches [53]. Representative dEST fasta sequences for each cluster were annotated to ko (KEGG Orthology) identifiers using the KEGG Automatic Annotation Server (KAAS) [41]. The dESTs were annotated by mapping to other well characterized invertebrate species; the fruit fly *Drosophila melanogaster* (Meigen, 1830) and the nematodes *Caenorhabditis elegans* (Maupas, 1900), *Caenorhabditis briggsae* and *Brugia malayi* (Brug, 1927). Finally, 932 KEGG identifiers could be annotated to the clusters.

### Jonckheere-Terpstra and Umbrella trend tests

The Jonckheere-Terpstra (JT) test is a non-parametric, rank-based test to analyse monotonic trends between groups [38,39]. The null-hypothesis states that *H*_0_:*G*_1_=*G*_2_=…=*G*_*k*_, where *G*_*i*_denotes either the distribution function or the median of the *i*-th of *k* ordered samples. The alternative hypothesis states: *H*_1_:*G*_1_≤*G*_2_≤…≤*G*_*k*_, where at least one of these inequalities strictly holds.

The Umbrella test is a generalisation of the JT test by Mack and Wolfe [40], which is used to test for trends with a peak also known as umbrella shaped data. The null-hypothesis *H*_0_ states that all samples were drawn from the same population *G*_*i*_with identical distribution: *H*_0_:*G*_1_=*G*_2_=…=*G*_*k*_. The alternative hypothesis states: *H*_1_:*G*_1_≤*G*_2_≤…≤*G*_*l*_≥*G*_*l* + 1_≥…≥*G*_*k*_where *l* is the peak position. For the JT test the implementation from the R-package SAGx was used [54], while the Umbrella test was implemented in R and will be made available in the package.

### Construction of metabolic reaction network

The complete KEGG reference pathways were downloaded as XML files [36] from http://www.genome.jp/kegg/xml/KGML_v0.7.1in December 2010. The KEGG pathways were loaded with the R package KEGGgraph [55] and processed using BioNet [35] and igraph [56]. All pathways were converted into graphs with compounds as nodes and reactions as edges. The graphs resulting from each pathway were combined into one supergraph. Subsequently, the graph was converted into an undirected graph and anomers of carbohydrates were fused to a single node (e.g. *α*- and *β*-D-glucose to D-glucose) containing all edges of the previously distinct nodes. Certain metabolites were removed which form non-specific shortcuts between the modules in the network. The excluded metabolites are: H^ + ^, H_2_O, P, ATP, NAD^ + ^, NADH, ADP, CO_2_, CoA, NADP^ + ^, NADPH, NH_3_, PP. Many of these metabolites serve as cofactors and they represent connections that are not involved in C-transfer, therefore they are less relevant for this purpose. They also correspond to highly-connected metabolites, whose concentrations are well-buffered and not likely to be informative about specific areas of metabolism. The considered metabolites are based on experiences from other analyses and we do not claim to provide an exhaustive list. We abstained from further preprocessing steps to reduce the model by eliminating unmeasured metabolites, as mentioned in [34,57]. In contrast, our algorithm enriches the significant regions during the optimization over node and edge scores by using the complete network. The constructed metabolic network consists of 3,658 nodes (metabolites) and 4,378 edges (reactions). The reference metabolic network will be made available in the package.

### Identifying functional modules in integrated networks

Integrated network analysis was performed by scoring the nodes and edges of the metabolite network derived from the trend test of the metabolites and log_2_ratios of dESTs. The analysis and search for optimal modules was performed as described in Dittrich *et al*. [21] with an exact algorithm termed heinz (heaviest induced subgraph). Briefly, the distribution of raw p-values from a statistical t-test can be considered as a mixture of signal and noise, where the signal component is modelled to be Beta(*a*,1) distributed [58], whereas the distribution of the noise component is by definition given as the uniform distribution. Fitting a Beta-uniform mixture (BUM) model, maximum-likelihood estimates for all model parameters can be obtained that are subsequently used to score the nodes of a biological network. The node score is given by the likelihood ratio of the signal to the noise component and can be adjusted by a threshold *τ*depending on a pre-selected false discovery rate (FDR)

(2) $$\;S^{\mathrm{FDR}}(x)=\log\left(\frac{ax^{a-1}}{a\tau^{a-1}}\right)=(a-1)\left(\log(x)-\log(\tau (\mathrm{FDR}))\right)\;.$$

Based on the node score, functional modules can be identified by finding the maximum-scoring subnetworks. Using an exact approach based on integer linear programming provably optimal and suboptimal solutions can be detected [21]. Additionally, extending the analysis of [21,35] edge weights can be integrated. The algorithm including integration of data, scoring of nodes and methods for network search and visualization are implemented in the open-source R package [35], available from http://bionet.bioapps.biozentrum.uni-wuerzburg.de/ and the Bioconductor project [59].

### Correspondence analysis

Correspondence analysis (CA) is a multivariate statistical method to visualize high dimensional data [60]. It was introduced for microarray data which contains thousands of genes together with multiple conditions by Fellenberg *et al.*[37]. For large-scale data it is desirable to reduce the dimensions of the data and look at the principal components which contain most information. This is accomplished by applying correspondence analysis and transforming the data by basis transformation, so that the principal axis are decreasingly ordered according to their information content. The data can then be visualized according to the first components. Likewise it can be applied to metabolite data with hundreds of metabolites. Correspondence analysis was performed throughout the study using the R package vegan [61].

### GO enrichment

For functional characterization of genes corresponding to the enzymes contained in robust components of the modules a gene ontology (GO) [62] term enrichment against the complete network was performed. This identifies the GO categories that are significantly overrepresented in a set of genes. The analysis was conducted using the R package GOstats [63].

## Competing interests

The funders had no role in study design, data collection and analysis, decision to publish, or preparation of the manuscript.

## Authors contributions

DB, TM, MD, GK did statistical and computational network analysis. TM and MD led and guided the study. GK, JK and ROS conceived and designed the experiments for the metabolite analyses. The GC-TOF-MS based metabolite profiles were performed and processed in the laboratory of JK. Tardigrades were sampled, extracted and shipped by SH in the laboratory of ROS. MAG and MF contributed the EST data. MAG performed base calling, pre-processing and clustering of EST sequences from the active and inactive stage. TD provided expertise on metabolic pathways. All authors participated in writing and approved the final version of the manuscript.

## Supplementary Material

Additional file 1 **Normalized metabolic profiles based on GC-MS for *M. tardigradum.*** Click here for file
